# Complete Genome Sequence of *Arcobacter* sp. Strain LFT 1.7 Isolated from Great Scallop (*Pecten maximus*) Larvae

**DOI:** 10.1128/genomeA.01617-16

**Published:** 2017-02-09

**Authors:** A. L. Diéguez, J. L. Romalde

**Affiliations:** Departamento de Microbiología y Parasitología, CIBUS-Facultad de Biología, Universidade de Santiago de Compostela, Santiago de Compostela, Spain

## Abstract

*Arcobacter* sp. strain LFT 1.7 was isolated from great scallop (*Pecten maximus*) larvae. Analysis of the 16S rRNA gene sequence showed that strain LFT 1.7 formed an independent lineage in the genus *Arcobacter*. The draft genome of LFT 1.7 was sequenced to determine the taxonomic position and ecological function of this strain.

## GENOME ANNOUNCEMENT

The genus *Arcobacter* was described by Vandamme et al. in 1991 (1) to accommodate two species of *Campylobacter*—namely, *C. nitrofigilis* and *C. cryaerophila*. This genus presents a global distribution and it has been associated with a great diversity of environments and hosts. Some members of *Arcobacter* are considered emergent enteropathogens and zoonotic agents (2), responsible for diarrhea and peritonitis in humans (3) or abortions and mastitis in animals (4). Consumption of contaminated water or food is considered the principal transmission route, but this is not clear. So, numerous studies have been carried out to estimate the prevalence of *Arcobacter* spp. in diverse environments and hosts such as water or mollusks. Due to these studies, the number of known species within this genus considerably increased in the past decade (http://www.bacterio.net/arcobacter.html).

The genome sequence of strain LFT 1.7 was obtained by Sistemas Genómicos (Valencia, Spain) using Illumina paired-end sequencing technology. The quality of the reads was analyzed using Trimmomatic version 0.32 (5). Genome assembly was performed using SPAdes version 3.6.1 (6), and QUAST (7). The resulting genome for LFT 1.7 consists of 451 contigs (>200 bp) of 3,617,612 bp and has a G+C content of 28.7%. The *N*_50_ contig size was 343,594 bp, with the largest contig being 1,211,507 bp. Automatic gene annotation was carried out by the Rapid Annotations using Subsystems Technology (RAST) server (8), and tRNAs were identified by tRNAscan-SE version 1.21 (9). The genome of LFT 1.7 contains 3,350 protein-encoding genes and 69 tRNAs. The annotation of the genome revealed 36 encoding regions of resistance to antibiotics, including beta-lactamase, and resistance to fluoroquinolones, as well as toxic compounds such as arsenic. This genome contains 74 genes related to nitrogen metabolism. Among them, 33 genes are involved in denitrification, with gene clusters encoding nitric oxide reductase, nitrous oxide reductase, and nitrite reductase. The other 41 genes are implicated in nitrate and nitrite ammonification, ammonia assimilation, dissimilatory nitrite reductase, or nitrosative stress. Also, 48 genes encoding bacterial hemoglobins and one flavohemoglobin were detected in the LFT 1.7 genome. These proteins confer protection from nitric oxide and nitrosative stress.

The genome sequence of this strain will help in the study of the heterogeneity of the genus *Arcobacter* and the ecological function of this bacterium in different environments.

### Accession number(s).

This whole-genome shotgun project has been deposited at DDBJ/ENA/GenBank under the accession number MKCO00000000. The version described in this paper is the first version, MKCO01000000.
